# Dataset on sinusoidally stiffened 3D printed steel plated structures

**DOI:** 10.1016/j.dib.2024.110193

**Published:** 2024-02-15

**Authors:** Jingbang Pan, Jie Wang, Mark Evernden, Yang Gu

**Affiliations:** Department of Architecture and Civil Engineering, University of Bath, Bath BA2 3GE, UK

**Keywords:** Additive manufacturing, Finite element modelling, Plate buckling, Stub column tests, Selective Laser Melting, Stiffening method, Sinusoidal waves, 316L stainless steel

## Abstract

The paper reports a series of experimental and numerical data of destructive stub column tests on additively manufactured steel parts stiffened by surface sinusoidal wave patterns. The specimens were made in 316L stainless steel and manufactured by selective laser melting (SLM). The experimental tests covered five tensile coupon tests, fourteen square hollow section (SHS) stub column tests and measurements of geometric imperfections of the stub columns. Numerical models incorporating the measured material and geometric properties were developed and analysed via GMNIA approach. The validity of the numerical models is demonstrated by their accurate replications of the load-end shortening responses of the tested specimens. The reported dataset will contribute to the stability design and characterisation of thin-walled steel plated structures with advanced stiffening patterns.

Specifications Table

**Table:** 

Subject	Civil and Structural Engineering
Specific subject area	Structural optimisation of thin-walled steel structures.
Data format	Raw, Analysed
Type of data	Table, Image, Figure, Spreadsheet
Data collection	Five tensile coupons and a total of fifteen stub columns were additively manufactured using the selective laser melting (SLM) technique. The test data was acquired from an experimental testing programme where fourteen square hollow section stub column tests were performed under compressive loading using an Instron hydraulic testing machine. The displacement of the stub columns was measured by linear variable differential transformers (LVDTs) while the load was read from the loading jack directly. Geometric data were obtained by laser scanning and measurement employing a vernier calliper. The numerical data were gathered from a finite element modelling programme run in ABAQUS 6.17, with input files generated by the programming language of Python 2.7.
Data source location	Institution: University of Bath Location: Bath, UK All experimental tests were carried out at the University of Bath, and all numerical work was completed using University computers and storage.
Data accessibility	Repository name: Figshare Data identification number: Direct URL to data: https://figshare.com/articles/dataset/Dataset_on_Sinusoidally_Stiffened_3D_Printed_Steel_Plated_Structures/25130072
Related research article	Pan, J., Wang, J., Evernden, M., Tian, Y., Chater, B. and Li, R., 2023. Experimental and numerical study of 3D printed steel plates stiffened by sinusoidal waves for enhanced stability. Engineering Structures, 293, p.116577. DOI: https://doi.org/10.1016/j.engstruct.2023.116577

## 1 Value of the Data

• These data can be useful in understanding and investigating the stability performance of thin-walled steel structures when stiffened by advanced stiffening shapes.
• Structural engineers and researchers can use the reported material and stub column test results and modelling techniques to develop and validate their numerical models on stainless steel plated structures under compressive loading.
• These data can be employed by other researchers as a benchmark to validate their numerical models, perform parametric studies and develop new design equations for the innovative structures with sinusoidal wave patterns.

## 2 Background

The experimental and numerical program was carried out to verify the efficiency of a novel plate stiffening method. The experiments covered a series of tensile coupon tests, stub columns and geometric dimension measurements, to provide initial inputs for numerical modelling. The validated numerical models were then used to develop a dataset with a wider range of geometric dimensions to help with finding the optimized stiffening geometry considering the effect of the associated manufacturing defects. The specimens with special 3D geometries were not able to be quickly made by traditional methods such as hot-rolling or cold-forming. Therefore, additive manufacturing using selective laser melting was chosen to protocol the proposed shape. The current paper describes the experimental testing and numerical modelling approaches involved in the study and provides a pool of the key data generated.

## 3 Data Description

**Fig. 1:**
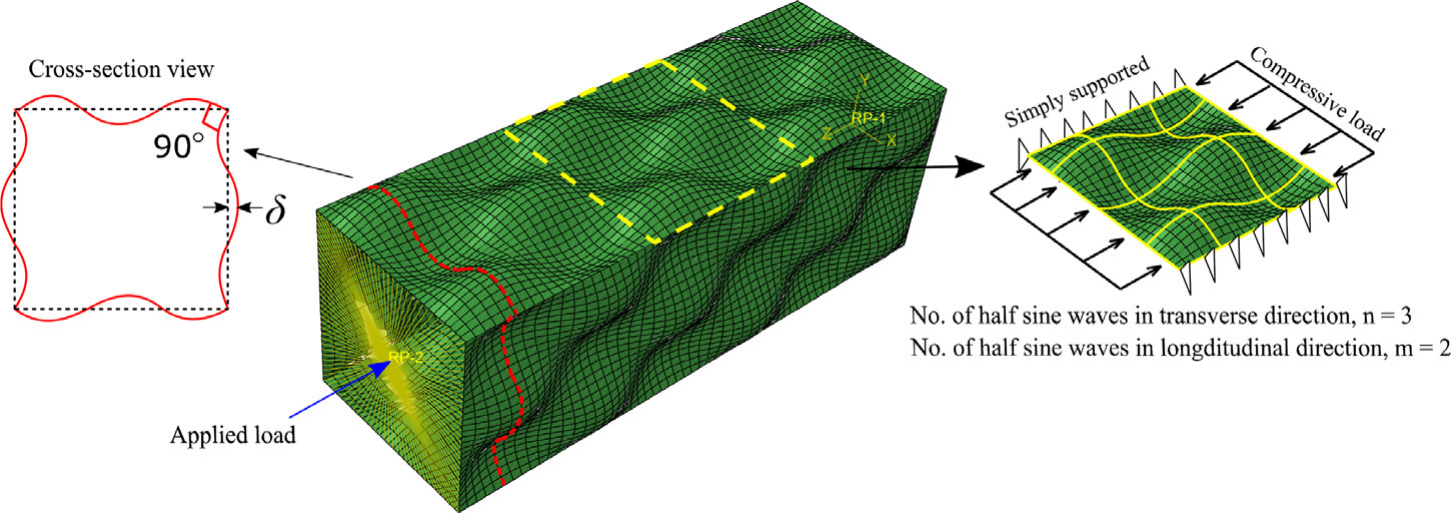
An illustration of the wavy stiffening approach and the stub column configuration [1].

The experimental and numerical data reported here is the key reference for the conclusions drawn in [1]. The experimental specimens were manufactured by selective laser melting and comprised of 5 tensile coupons and 14 square hollow section stub columns in 316L stainless steel. While the tensile coupons were tested to obtain the material stress-strain relationship of additive manufactured stainless steel material, the stub column specimens were manufactured with pre-defined surface wave patterns to verify a novel stiffening approach experimentally and provide a reference for the further numerical study. The tensile coupons were named as coupon x, with x being the number of the coupon. The stub columns were manufactured in the shape of square hollow sections, assembling four equal and mutually supported plates to represent the boundary condition for ‘stiffened’ plates. The flanges of the stub columns were printed in patterns of 2-direction sinusoidal waves, with n and m representing the numbers of half-sinusoidal waves in the transverse and longitudinal directions of the stub columns in a squared zone, as illustrated in Fig. 1. The stub columns contained three cross-section dimensions (SHS 50 × 50 × 2, SHS 60 × 60 × 2 and SHS 70 × 70 × 2), three waveforms (n2m1, n3m1 and none) and two wave amplitudes (2mm and 3mm), giving a total of 15 specimens. It should be noted that 1 specimen was used for residual stress measurement therefore only 14 stub column tests were carried out.

**Fig. 2:**
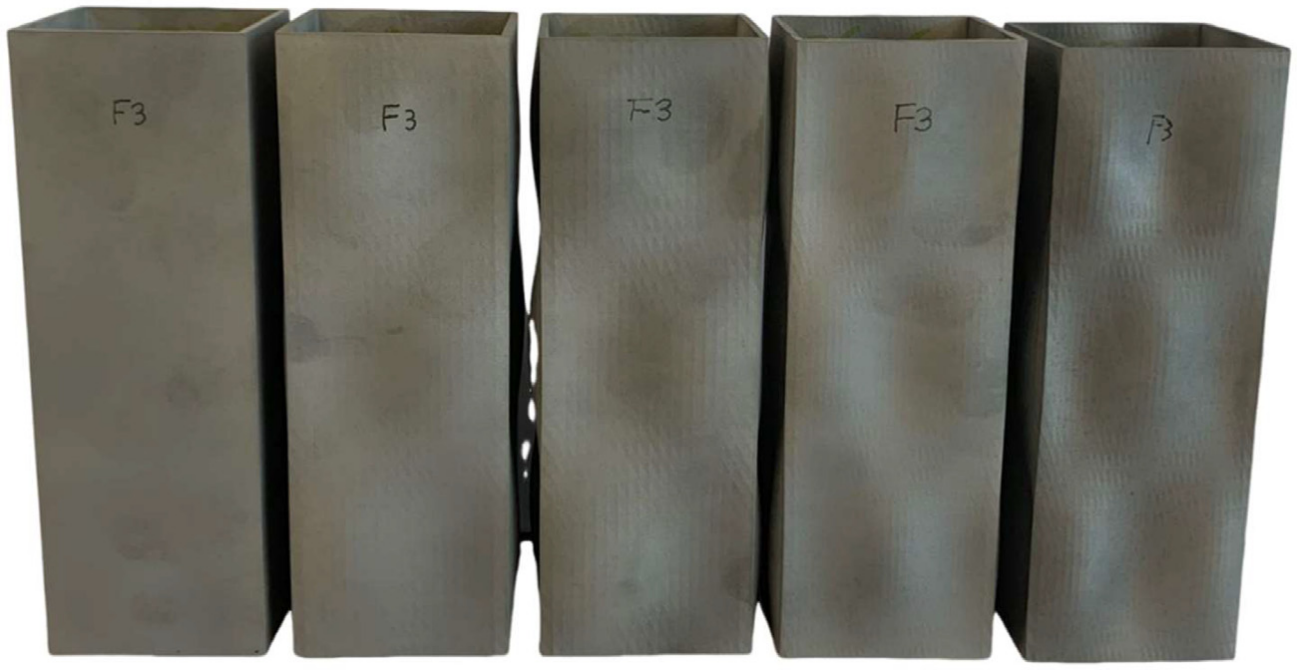
SHS 60 × 60 × 2 stub columns with different wave patterns before testing (from left to right: 60n0m0, 60n2m1-2, 60n2m1-3, 60n3m1-2 and 60n3m1-3).

**Fig. 3:**
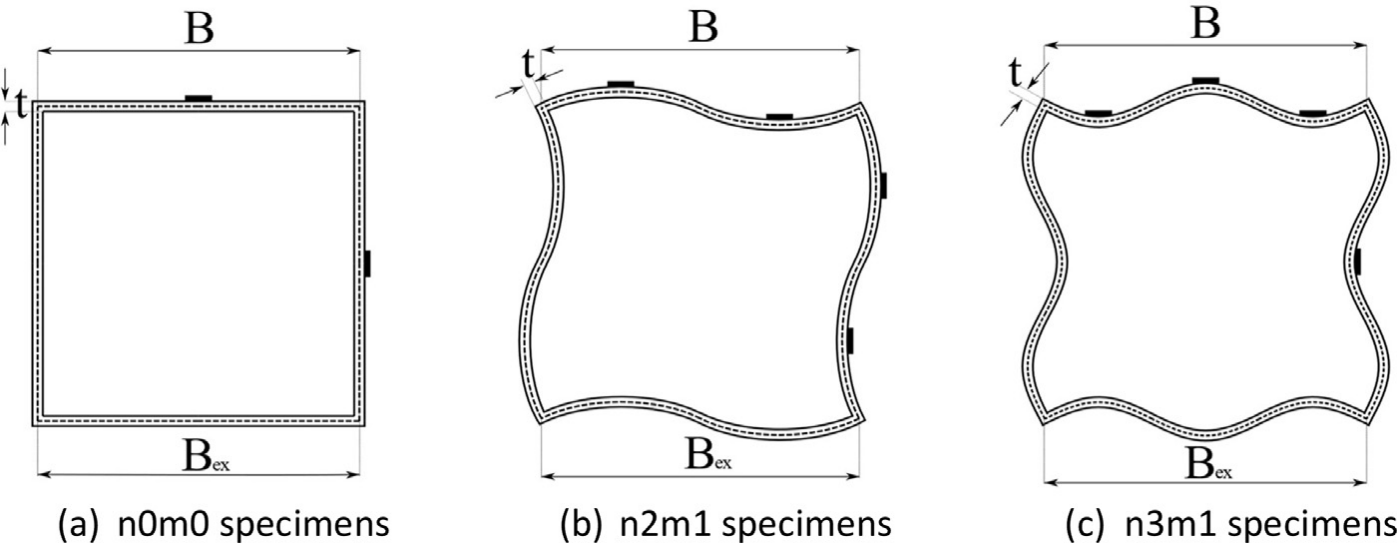
Notations of dimensions and strain gauge locations (width to thickness ratio is 25 in figures and subject to change for varying cross-section sizes) [1]

A photo of SHS 60 × 60 × 2 specimens prior to the stub column tests is given in Fig. 2. From the cross-sectional view in Fig. 3, the corners of the specimens remain 90 degrees between the adjacent flanges, and the external width *B*_ex_ and the central-line width *B*, measured as *B*_ex_ minus the wall thickness *t*, are labelled. The geometric dimensions of the 15 stub columns were obtained by 3D laser scanning, which were in .txt format, provided in the file named “**Summary of laser scanned point clouds of SHS with varying wave patterns**”. The three columns of data in each file represent the geometric measurements along x-, y- and z- axes, which were captured by laser scanner ROMER ABSOLUTE ARM 7535 with a precision of 0.0406 mm. The measured point clouds were processed against their digital model counterparts using the geometric metrology software Spatial Analyser. Constrained by the internal dimensions of the hollow section stub columns and characteristics of Romer Absolute Arm laser scanner, only the outer width, *B*_ex_, of each specimen was measured at the one-third positions along the length. The geometric properties including dimensions, amplitude and the average geometric deviation against digital model *w*_0,mean_ (in mm) and the maximum geometric deviation *w*_0,max_ (in mm), are summarised in the excel file “**Summary of test data of SHS with different wave patterns.xlsx**” in the data repository. In this file, the specimen name consists of the nominal external dimension of the stub column (e.g. 50 for SHS 50 × 50 × 2 cross-section), followed by the wave pattern and the wave amplitude (e.g. 50n2m1-2).

**Fig. 4:**
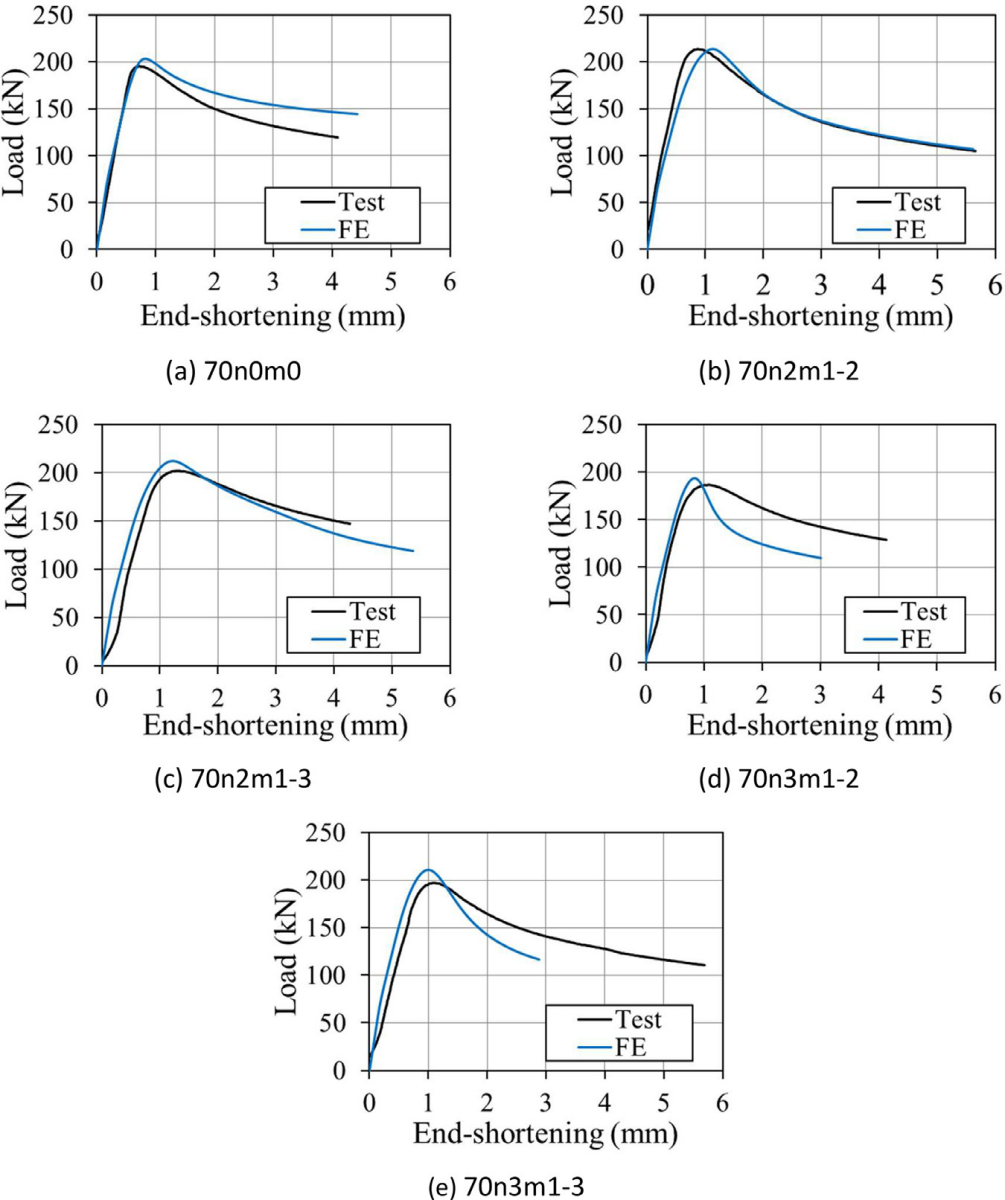
Experimental and numerical load vs axial displacement curves of wavy stub columns with *B* = 70 mm but different wave patterns.

The measured material stress-strain response of the tested 316L stainless steel tensile coupons are reported in [1], and the key material properties are shown in the excel file “**Summary of test data of SHS with different wave patterns.xlsx**”. It includes the measured Young's modulus, *E*_0_, yield strength, *σ*_0.2_, the failure stress *σ*_u,_ and strain hardening parameters *n*_0_, *n*_0.2,1.0_ and *n*_0.2,u_, as used in a three-stage model proposed by Hradil et al. [2] to reconstruct the stress-strain response used in further finite element analysis. The true stresses and strains, translated from the reconstructed stress-strain relationships using the average material properties [3], were employed in the finite element analysis. The stress-strain data of tensile coupon tests and load-axial displacement data of stub column tests are presented in the file “**Experimental stress-strain and load-disp of SHS stub columns.xlsx**”. The FE GMNIA calibrations of the stub column tests are shown in “**Summary of FE calibrations of SHS stub column tests.xlsx**”, where the ultimate strengths obtained from the FE models are compared with the corresponding tested values, and in “**Numerical load-disp of SHS stub columns.xlsx**”, where the FE load-displacement responses are given. Comparisons between the test and FE load-displacement curves of the stub columns are partially presented in Fig. 4, indicating the accuracy of the FE models developed.

## 4 Experimental Design, Materials and Methods

### 4.1 Test Data

The experimental tests involved five tensile coupon tests and fifteen stub column tests. The coupons were made in the same dumbbell shapes and tested in an Instron 50 kN loading frame, with a tension rate of 0.00007 s^−1^ pre-yielding and 0.00024 s^−1^ post-yielding, as stated in BS EN ISO 6892-1 [4]. The monitored variables covered the load read from the machine, the strain development measured by an 8-mm clip gauge and two strain gauges fixed to the mid-height of the coupon.

**Fig. 5:**
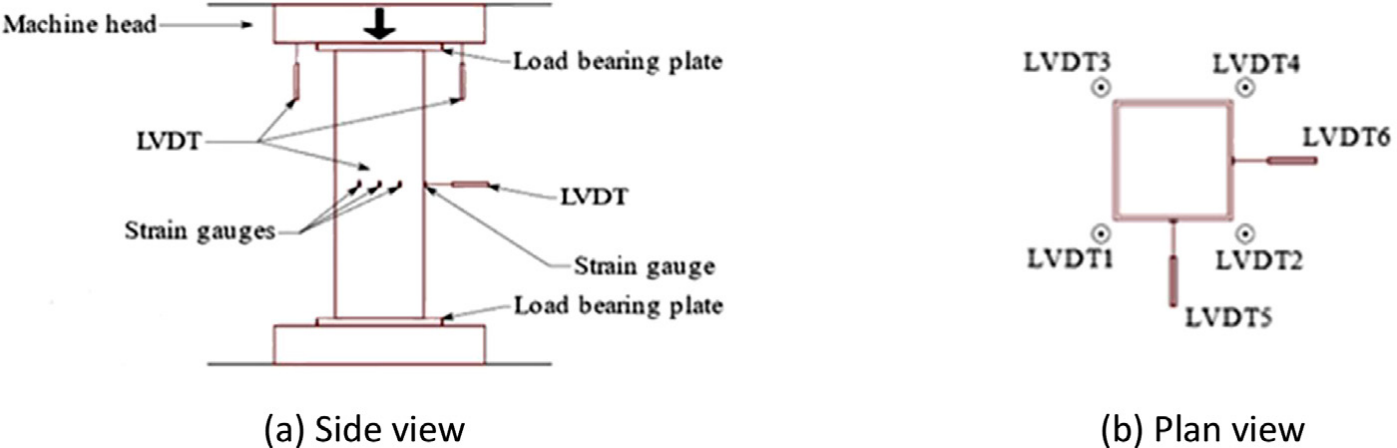
Schematic views of stub column test set-up [1].

All the stub column tests were performed in a 2000kN DARTEC hydraulic testing machine, which employed compressive stresses to the stub column positioned between two parallel loading plates, as is shown in Fig. 5. Four LVDTs were contacted with the top load-carrying end plate to measure the vertical displacement of the stub columns during testing. Since one or two repeated patterns exist across the four surfaces of a stub column, strain gauges were attached to the mid-height locations of the stub columns on two adjacent surfaces to investigate the development of in-plane strains, while a single LVDT was in contact with the centroid of each face to measure the out-of-plane displacement. The axial load was applied with displacement control at a rate of 0.2 mm/min, and the loading history was recorded by the testing machine. All the monitored variables were recorded at one-second intervals.

### 4.2 Numerical Data

**Fig. 6:**
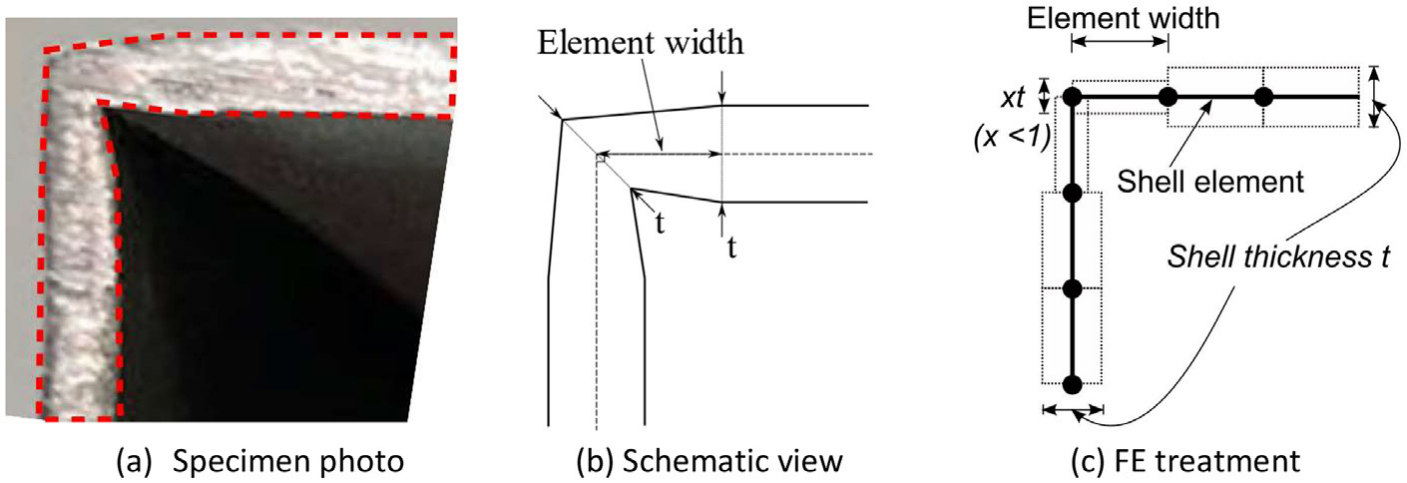
Description of corner element thickness reduction [1].

The numerical data were generated from a finite element modelling program using the software package ABAQUS 6.17. The stub column models were discretised with the four-node and reduced integration shell element (S4R), which is appropriate for the modelling of thin-plated structures. An element size of 10 elements per half-sinusoidal wave was adopted, giving different mesh sizes for different wave patterns, but indicating good accuracy from a mesh sensitivity study [5]. The engineering stress-strain relationship, as gathered from the tensile coupon tests, was transformed into the true stress-strain response for finite element modelling. The boundary conditions of the shell-based stub columns were fixed at both ends, with the end section edges coupled to a reference point on each side with zero eccentricity. Since the stub column specimens were 3D printed accordingly digital models built by rendering the thickness of the shell element, the rendered model has reduced thickness in the corner region, as illustrated in Fig. 6. This has been considered in the finite element model calibration by using reduced thickness (0.75*t*) at the corner region (Fig. 6c). Input files used in the simulation of 70 × 70 × 2 patterns were covered in “**ABAQUS Input data for the simulation of 70 × 70 × 2 specimens**” in data repository. An initial geometric perturbation in the first eigenvalue buckling mode (equivalent to the n1m1 wave pattern) was applied to each numerical model to reflect the actual structural behaviour. From an imperfection sensitivity study, it was found that different wave patterns have different sensitivity to imperfections [1], therefore different perturbation amplitudes were adopted for each model, as detailed in “**Summary of FE calibrations of SHS stub column tests.xlsx**”.

## Limitations

Not applicable.

## Ethics Statement

The authors have read and followed the ethical requirements for publication in Data in Brief and confirm that the current work does not involve human subjects, animal experiments, or any data collected from social media platforms.

## CRediT authorship contribution statement

**Jingbang Pan:** Data curation, Formal analysis, Investigation, Software, Methodology, Writing – original draft. **Jie Wang:** Conceptualization, Project administration, Resources, Data curation, Formal analysis, Software, Methodology, Supervision, Writing – review & editing. **Mark Evernden:** Conceptualization, Project administration, Resources, Methodology, Supervision. **Yang Gu:** Software, Methodology.

## Data Availability

Dataset on Sinusoidally Stiffened 3D Printed Steel Plated Structures (Original data) (Figshare).
